# Poxvirus immunotherapies in combination with immune checkpoint inhibitors synergize to eliminate tumors in a mouse tumor model

**DOI:** 10.1186/2051-1426-1-S1-P72

**Published:** 2013-11-07

**Authors:** Susan P Foy, Ryan B Rountree, Stefaine J Mandl, Joseph Cote, Tracy dela Cruz, Evan Gordon, Erica Trent, Alain Delcayre

**Affiliations:** 1 Research and Development, Bavarian Nordic, Inc., Mountain View, CA, USA

## 

Bavarian Nordic, Inc. is developing poxvirus-based cancer immunotherapies. These therapies employ vaccinia-, Modified Vaccinia Ankara (MVA)-, and fowlpox-based vectors that were engineered to express one or more tumor-associated antigens (TAA). These vectors are used alone or in prime-boost strategies to generate an active immune response against a variety of cancers. The lead candidate PROSTVAC® employs a prime-boost strategy using vaccinia and fowlpox expressing PSA and TRICOM(TM) and is currently in a global Phase III clinical trial (PROSPECT) for castration-resistant metastatic prostate cancer. Other products such as recombinant MVA-BN® expressing HER2 have been tested in early phase clinical trials for the treatment of breast cancer. To further enhance the anti-tumor efficacy of the poxvirus-based immunotherapy, the present preclinical study focuses on combining the breast cancer candidate therapy MVA-BN® HER2 with a monoclonal antibody that blocks the activity of CTLA-4, an immune checkpoint protein that down-regulates T cell activation. In the CT26-HER2 experimental lung metastasis model, the median survival time increased from 30 days in untreated mice to 49.5 days with MVA-BN® HER2 treatment while anti-CTLA-4 treatment by itself showed little survival benefit (median survival 35 days). In contrast, MVA-BN® HER2 in combination with anti-CTLA-4 significantly increased the survival to greater than 100 days (p<0.0001) in more than 50% of the mice. At 100 days, the lungs of the surviving mice were examined and there were no visible tumors. Furthermore, phenotypic analysis was performed on tumor infiltrating lymphocytes. At day 25, an increase in the number of regulatory T cells (CD4+ FoxP3+) was observed in the lungs of untreated and anti-CTLA-4 treated mice which correlated with increased pulmonary tumor burden. Effector cells (CD127+ KLRG1+/-) increased with MVA-BN® HER2 treatment and were further increased in combination with anti-CTLA-4. The inducible co-stimulatory molecule (ICOS) also increased with MVA-BN® HER2 treatment and was enhanced with anti-CTLA-4 treatment on both CD4+ and CD8+ T cells. Overall these animal studies demonstrate that enhanced efficacy and synergistic activity of poxvirus immunotherapies can be achieved in combination with anti-CTLA-4 and provide insight into the phenotype of the immune cells. Additional checkpoint inhibitors in combination with poxvirus vectors are being actively studied and will be discussed in greater detail.

